# Immune Deregulation in Sepsis and Septic Shock: Reversing Immune Paralysis by Targeting PD-1/PD-L1 Pathway

**DOI:** 10.3389/fimmu.2020.624279

**Published:** 2021-02-17

**Authors:** Yuki Nakamori, Eun Jeong Park, Motomu Shimaoka

**Affiliations:** Department of Molecular Pathobiology and Cell Adhesion Biology, Mie University Graduate School of Medicine, Mie, Japan

**Keywords:** sepsis - diagnostics, immunomodulation, immunoparalysis, PD-1, PD-L, immune checkpoints inhibitors, artificial intelligence, machine learning

## Abstract

Sepsis remains a major problem for human health worldwide, thereby manifesting high rates of morbidity and mortality. Sepsis, once understood as a monophasic sustained hyperinflammation, is currently recognized as a dysregulated host response to infection, with both hyperinflammation and immunoparalysis occurring simultaneously from the earliest stages of sepsis, involving multiple organ dysfunctions. Despite the recent progress in the understanding of the pathophysiology underlying sepsis, no specific treatment to restore immune dysregulation in sepsis has been validated in clinical trials. In recent years, treatment for immune checkpoints such as the programmed cell death protein 1/programmed death ligand (PD-1/PD-L) pathway in tumor-infiltrating T-lymphocytes has been successful in the field of cancer immune therapy. As immune-paralysis in sepsis involves exhausted T-lymphocytes, future clinical applications of checkpoint inhibitors for sepsis are expected. In addition, the functions of PD-1/PD-L on innate lymphoid cells and the role of exosomal forms of PD-L1 warrant further research. Looking back on the history of repeatedly failed clinical trials of immune modulatory therapies for sepsis, sepsis must be recognized as a difficult disease entity for performing clinical trials. A major obstacle that could prevent effective clinical trials of drug candidates is the disease complexity and heterogeneities; clinically diagnosed sepsis could contain multiple sepsis subgroups that suffer different levels of hyper-inflammation and immune-suppression in distinct organs. Thus, the selection of appropriate more homogenous sepsis subgroup is the key for testing the clinical efficacy of experimental therapies targeting specific pathways in either hyperinflammation and/or immunoparalysis. An emerging technology such as artificial intelligence (AI) may help to identify an immune paralysis subgroup who would best be treated by PD-1/PD-L1 pathway inhibitors.

## Introduction

Sepsis remains an ongoing threat to human health worldwide. Sepsis is one of the leading causes of death in intensive care units (1, 2). The World Health Organization (WHO) has recommended that sepsis be recognized as a Global Health Priority (3). Although there has been an overall improvement in clinical outcomes globally, which seems due to improved treatment practices by disseminating and complying with the Surviving Sepsis Campaign guidelines (4) during the past few decades (5), mortality rates remains unbearably high, reaching 25%–30% for sepsis, and up to 40%–50% in cases of septic shock, though there are some differences depending on the country (6, 7). To put it simply, the central pathophysiology of sepsis is dysregulated host response to infection (8). The concept of dysregulation includes not only excessive inflammation, but also immunosuppression. Moreover, hyper-inflammation and immunoparalysis can exist concomitantly from the very onset of sepsis. A previous biphasic concept that the immunosuppressive late phase follows the hyperinflammatory early phase has now been outdated (9). The purpose of this review is to describe the underlying mechanisms of the immune deregulation in sepsis, to summarize the failed history of clinical trials testing therapeutic agents for host immune responses in sepsis, and to specifically address treatments aimed to reverse the immunosuppression, focusing in particular on the programmed cell death protein 1/programmed death ligand (PD-1/PD-L) pathway on T cells. In addition, the functions of the PD-1/PD-L pathway on innate lymphoid cells and PD-L1 present on exosomes are discussed as characteristic aspects of sepsis in this review. In the last section, we discuss the application of artificial intelligence (AI) for subgrouping of septic patients and selecting the appropriate patients as the most promising approach for achieving a breakthrough in new treatments for sepsis. If a revolution in sepsis treatment is to be realized, new concepts and new methods are absolutely essential. This means immunomodulation and AI.

## The Clinical Definition of Sepsis

Although the breadth and depth of our knowledge about the pathophysiology underlying sepsis has increased dramatically in recent years thanks to the expansion of biomedical research publications, there has been only limited progress in treatment in terms of interventions for the essential host response rather than supportive care such as mechanical ventilation strategies, nutrition, and PADIS (Pain, Agitation, Delirium, Immobility, and Sleep) management. In addition, chronic persistent inflammatory-immunosuppressive syndrome has emerged as a new topic regarding survival in wake of acute phases of sepsis. Quality of life after surviving initial septic insult and discharge remains poor. Thus, sepsis ranks among today’s leading medical, economic, and social issues.

Historically, the term “sepsis” has been used extensively, although until about 1990 there was no clear definition. In 1991, the ACCP/SCCM (American College of Chest Physicians/Society of Critical Care Medicine) Consensus Conference defined sepsis as Systemic Inflammatory Response Syndrome (SIRS), which was caused by microbial infections (10). The SIRS case must meet two or more of the following four criteria; tachypnoea, tachycardia, abnormal body temperature and/or abnormal white blood cell count or >10% presence of immature white blood cell forms. This definition emphasizes the concept that systemic inflammation is the key to sepsis, and therefore mandates physical examinations or laboratory parameters that address hyper inflammation. Until the advent of the Third International Consensus Definitions for Sepsis and Septic Shock (Sepsis-3), sepsis was largely understood as a systemic inflammatory syndrome. In 2016, Sepsis-3 defined sepsis as life-threatening organ dysfunction caused by a dysregulated host response to infection (11). This new definition showed that the past characterizations were excessively focused on inflammation. Septic shock was defined as a condition requiring continuous administration of vasopressors to maintain mean arterial blood pressure, despite initial fluid resuscitation. In Sepsis 3, the term “severe sepsis” was eliminated. This meant that the pathological condition of sepsis, which causes organ damage as a result of infection, was in itself a very serious pathology.

## Immune Deregulation; The Central Pathophysiology of Sepsis/Septic Shock

Sepsis constitutes a dysregulated host response to infection. Hyper-inflammation and the immunoparalysis can exist concomitantly from the onset of sepsis. In most cases, infections are contained and eventually cured by the cooperations by the immune system, antibiotics, and source control/drainage, thereby restoring to normal homeostasis. However, infection can progress to sepsis when a dysregulated host response persists. The time course of sepsis was previously thought to consist of an initial hyperinflammatory state that transitioned to a hypoinflammatory state, eventually leading to prolonged and significant immunosuppression. In terms of paired words, systemic inflammatory response syndrome (SIRS) and compensatory anti-inflammatory response syndrome (CARS) symbolize this paradigm (12).

**Figure 1:**
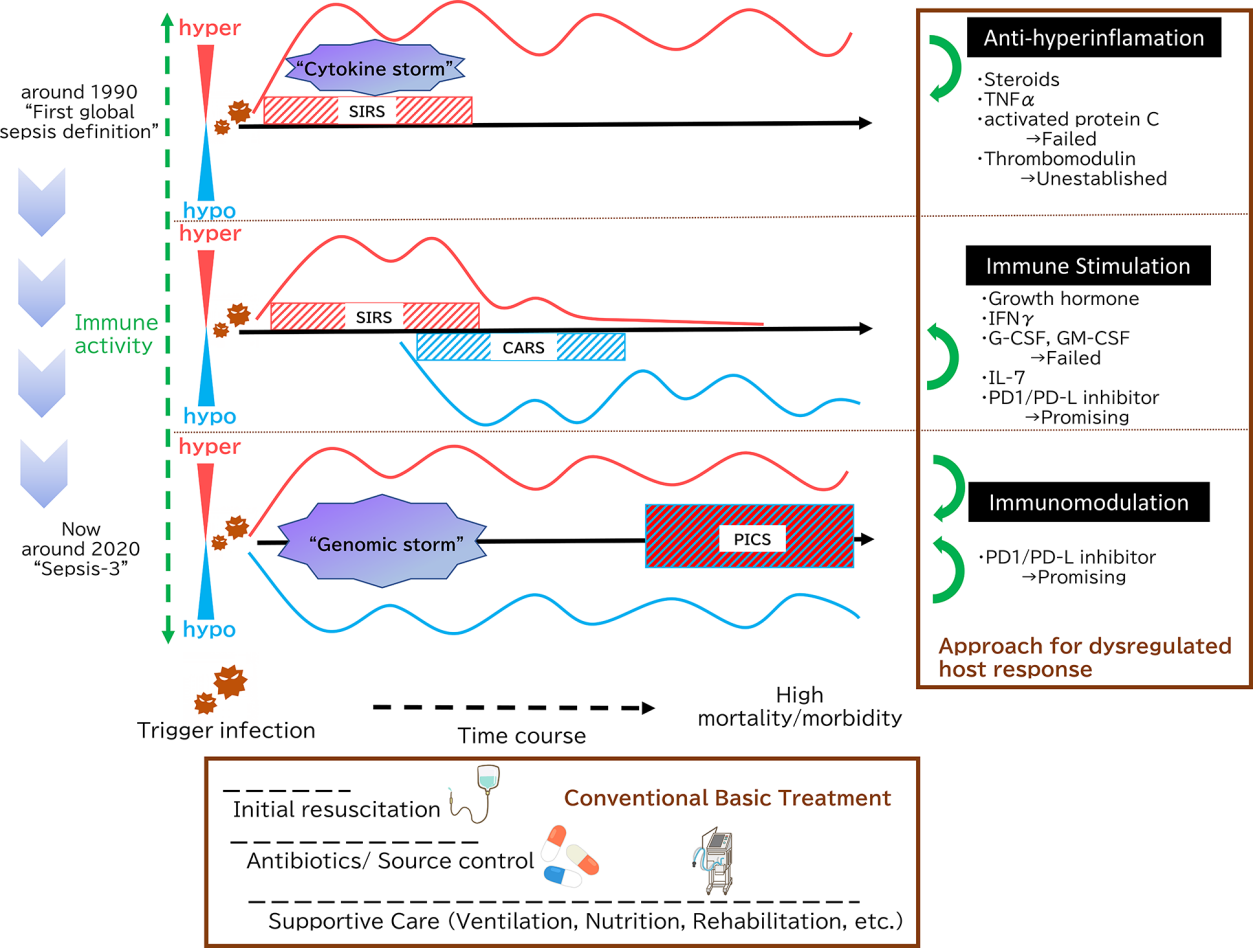
Paradigm shift in sepsis. In 1991, sepsis was defined as Systemic Inflammatory Response Syndrome (SIRS), which was caused by microbial infections. This definition emphasizes the concept that systemic inflammation is the key to sepsis. During the initial phases of sepsis, inflammation originating in the innate immune system is enhanced by multiple pathways as “cytokine storm”. Then, a new theory has since emerged positing that immunosuppression following initial hyperinflammation, eventually leading to prolonged and significant immunosuppression is the key pathophysiology. In terms of paired words, SIRS and compensatory anti-inflammatory response syndrome (CARS) symbolize this paradigm. In 2016, The Third International Consensus Definitions for Sepsis and Septic Shock (Sepsis-3) defined sepsis as life-threatening organ dysfunction caused by a dysregulated host response to infection. Both pro-inflammatory and anti-inflammatory genomic storms occur beginning in the earliest stages of infection, and the balance between the two determines whether clinically over-inflammation or immunosuppression then occurs. Persistent Inflammation, Immunosuppression, and Catabolism Syndrome (PICS) is an intriguing concept from the integrated point of view, which contends that SIRS and its counterpart CARS do not exist independently; rather, both are occurring simultaneously. As shown in the Surviving Sepsis Campaign guidelines, initial fluid resuscitation, earlier antibiotic administration and supportive care such as mechanical ventilation strategies, nutrition and PADIS (Pain, Agitation, Delirium, Immobility, and Sleep) management are key of sepsis management. With the development of these conventional basic treatment, there is an overall clinical outcome improvement, but mortality of sepsis still reaches high. Therefore, additional treatment method that targets the underlying essence of sepsis has been expected. At first, as sepsis was essentially understood as hyperinflammation, many anti-inflammation approaches were tried. However, none have demonstrated that those sepsis treatment strategies are effective. Then, focusing on the immunoparalytic aspect of sepsis, immune stimulation represents a new strategy for targeting sepsis. There are some promising molecules, among them PD-1/PD-L inhibitors, which can not only reverse immuostimulation but act as immunomodulation, are highly expected.

Recent studies have shown that inflammation and immunosuppression occur simultaneously but not sequentially. Both pro-inflammatory and anti-inflammatory cytokine storms occur during the earliest stages of infection, and the balance between the two determines whether clinically over-inflammation or immunosuppression then occurs (13). (**Figure 1**) Analyses of leukocyte gene expression in patients with severe sepsis have revealed that both the inflammatory response and the expression of immunosuppression-related genes occur at the same time, immediately after sepsis onset. In fact, the more severe a patient’s case is, the higher the immunosuppression-related gene expression level (14). Excessive inflammation is not the only cause of death in sepsis. Similarly, immunosuppression is not the only cause of sepsis-related death. A mixture of the two is responsible for the difficulty of treating sepsis and its overall poor prognosis.

There is also a concept that sepsis constitutes what has been termed Persistent Inflammation, Immunosuppression and Catabolism Syndrome (PICS) (15). This is an intriguing concept from the integrated point of view, which contends that SIRS and its counterpart CARS do not exist independently; rather, both are occurring simultaneously. To summarize, in sepsis, hyper-inflammation and immunoparalysis coexist from the early to the late stages of the pathological condition, which is evident from recent basic researches and clinical recognitions.

### Sepsis Induced Hyper-Inflammation

Knowing that inflammation and immunosuppression occur simultaneously from the earliest stages of infection, hyperinflammation remains a hallmark of sepsis. During the initial phases of sepsis, acute inflammation originating from the activation of the innate immune system is triggered and enhanced by multiple pathways. Key pro-inflammatory responses contain various biological systems including complement system, coagulation system, platelet, vascular endothelium, neutrophil extracellular traps (NETs), and many kinds of immunity-oriented cells. These pro-inflammatory systems interact in a highly intricate manner. The word “cytokine storm” is a suitable description and well worth underscoring.

After infection, invading pathogens are immediately recognized by the host innate immune system. Innate immune cells such as neutrophils and macrophages, whose primary function is immune surveillance, recognize pathogen-associated molecular patterns (PAMPs) derived from microorganisms *via* pattern recognition receptors (PRRs) (16). PRRs include Toll-like receptors (TLRs), nucleotide-binding oligomerization domain-like receptors (NLRs), retinoic acid-inducible gene-like receptors (RLRs) and C-type lectin receptors (CLRs) (8, 16). PRRs recognize not only PAMPs derived from microorganisms, but also damage-associated molecular pattern molecules (DAMPs or Alarmin) (17). The latter are derived from the host’s own cells. The fact that PRRs recognize various PAMPs and DAMPs explains similar clinical appearances presented by critically ill patients, regardless of the trigger infections caused by various pathogens originating at various organs (18).

NF-κB and interferons (IFNs) pathways are two major signaling cascades to activate innate immune systems. These intracellular signaling cascades generate pro-inflammatory mediators by promoting gene transcription beginning within a few minutes of PRRs recognizing PAMPs or DAMPs. In the NF-κB pathway, the binding of PAMPs to TLR4 triggers the recruitment of the myeloid differentiation primary response gene 88 (MyD88), which acts as an adopter protein, and interleukin-1 receptor-associated kinases 1 and 4 (IRAK1, 4), act as signaling molecules. Then these signaling molecules form a complex with tumor necrosis factor (TNF) receptor-associated factor 6 (TRAF6). This cascade further activates the downstream TGF-β-activated kinase and inhibitor of nuclear factor kappa-B kinase (IKK), which then regulate NF-κB. NF-κβ, which is usually present in the cytoplasm, migrates to the nucleus where it activates targeted genes coding pro-inflammatory cytokines like TNF and IL-1. These cytokines then re-initiate signaling cascades to activate or produce other inflammatory cytokines and chemokines inter- or intra-cells in the innate immune response (19). IFNs are a group of signaling proteins that have diverse effects on the innate immune system (20). By binding its receptor, IFN starts the signaling cascade, leading to the expression of interferon-stimulated genes (ISGs) and IFN gamma-activated site (GAS) genes. In these inflammation cascades, small ubiquitin-like modifier (SUMO) molecules play an important role by binding to target proteins and modulating their function. In the NF-kB pathway, SUMO proteins play a dual role of promoting and limiting excessive activation of them. That is, SUMO-1 binds to the TRAF family member-associated NF-kappa-B activator while SUMO-3 binds to IKK-γ, which is one of the NF-κB essential modulators accelerating the NF-kB pathway. On the other hand, sumoylation of IκBα by SUMO-1 limits excessive activation (21). This bidirectional effect of SUMO can be observed in the IFN pathway, as well. For example, SUMO has both promoting and inhibiting effects on antiviral interferon regulatory transcription factor-3 (IRF-3) activity, through sumoylation and desumoylation, *via* peptidyl-prolyl cis/trans isomerase (Pin1) (21, 22).

### Failures of Anti-Inflammatory Therapies in Clinical Trials

Most basic and clinical research efforts on sepsis has focused on suppressing hyperinflammation. Numerous experiments involving animal sepsis models have shown that the suppression of specific inflammatory cascades improved outcomes (23–25). Over the last few decades more than one hundred clinical studies have sought to suppress excessive inflammation in sepsis by targeting PRRs, PAMPs, cytokines and various mediators, however, none have convincingly proved the clinical effectiveness for the treatment of sepsis in clinical settings. Here, we show representative clinical trials focusing on adrenal insufficiency, pro-inflammatory cytokines and immunothrombosis.

Relative adrenal insufficiency, also known as critical illness-related corticosteroid insufficiency, occurs in critically ill patients (26). Corticosteroids have been used for the purpose of early recovery from shock, rather than for reducing mortality, as indicated in the guideline published by the Society of Critical Care Medicine (SCCM) and the European Society of Intensive Care Medicine (ESICM) in 2017 (27). Glucocorticoids affect both innate and adaptive immunity; they inhibit the maturation, differentiation, and proliferation of leukocytes, including myeloid cells and lymphocytes. Glucocorticoids have an anti-inflammatory effect by producing anti-inflammatory proteins and inhibiting pro-inflammatory proteins. Glucocorticoids bind to glucocorticoid receptor (GR), which is a transcription factors belonging to the nuclear receptor superfamily, and GR translocates to the nucleus. The GR-glucocorticoid complex inhibits the production of pro-inflammatory proteins by sequestering NF-κB into the cytoplasm. It also promotes the production of annexin 1, which inhibits the expression of phospholipase A2. The functional differences between mineralocorticoid and glucocorticoid are still under investigation, although there are many overlapping aspects. It is worth noting that the expression of GR is higher in the immune system than that of mineralocorticoid receptor (MR), but MR and GR are similarly expressed in the cardiovascular system (28). Corticosteroids also have the disadvantages of elevating blood glucose levels and increasing catabolism. Therefore, an investigation as to whether or not corticosteroids can improve patient prognosis has long been needed. However, two large randomized controlled trials (RCTs) published in 2018 failed to produce consistent results. The APROCCHS (Activated Protein C and Corticosteroid for Human Septic Shock) trail, which compared activated protein C, low does corticosteroid, their combination, and placebo, demonstrated that sepsis patients treated with corticosteroid showed a significantly improved 90-day mortality rate (29), while the ADRENAL (Adjunctive corticosteroid treatment iN critically ilL patients with septic shock) trial reported no significant benefits at all (30). There are many points worth discussing regarding these two trials; e.g., the differences in sepsis severity between them and the different medication regimens using fludrocortisone, in addition to hydrocortisone, or not. In the end, however, the role of steroids in improving the prognosis of sepsis patients remains inconclusive.

Another approach involves suppressing inflammatory cell signaling proteins, which seems to have a direct effect on the hyper-inflammatory states. Among inflammatory proteins, TNF-α is a pro-inflammatory cytokine that has been shown to increase in the blood after endotoxin administration (31). Moreover, administration of TNF-α induces a biological reaction similar to those of sepsis (32, 33). Therefore, it is reasonable to hypothesize that inhibition of TNF-α represents a potential treatment option for sepsis in patients. Preclinical studies using animal model experiments have shown favorable results: a protective effect against sepsis (34, 35). However, a single dose of tumor necrosis factor receptor-Fc (TNFR-Fc), which is a fusion protein comprised of the TNFR extracellular domain and the Fc region of the human immunoglobulin heavy chain, injected into 141 patients with septic shock generated no improvements in patient prognosis. On the contrary, it unexpectedly increased mortality in a dose-dependent manner (36). Thus, this potential therapeutic agent can actually worsen the prognosis. The usefulness of TNFR-Fc was demonstrated later in rheumatoid arthritis (37). In both sepsis and rheumatoid arthritis, the central role of TNF in dysregulation of the immune system leading to hyperinflammation were assumed to be the essence of the pathology. However, insights gained from this clinical trial on TNF-α inhibition does not support a simple concept of hyper-inflammation induced by TNF driving the pathogenesis of sepsis.

Immunothrombosis refers to the crosstalk that occurs between coagulation and the immune system (38, 39). Thrombus formation may play an important role in preventing the spread of infection from local sites to the entire body. However, excessive coagulation and platelet activation can induce immune cell recruitment and inflammation. Overactivated platelets cause not only vascular occlusion in micro vessels, but also tissue injury due to the release of platelet-derived microparticles (40). Moreover, these platelets also cause excessive consumption of coagulation factors, resulting in so-called disseminated intravascular coagulation (DIC). Furthermore, inflammation induced the expression of tissue factor on monocyte, thereby triggering the coagulation cascades that lead to the platelet activation (41). With this theoretical background, the suppression of excessive coagulation related to sustained inflammation can be an effective treatment. Activated protein C, which is converted from protein C *via* a complex of thrombin and thrombomodulin, promotes fibrinolysis and suppresses thrombus and inflammation during inflammatory activation in sepsis (42). In addition, activated protein C has been shown to suppress inflammation by directly acting on neutrophils to inhibit integrin activation and NETs formation (43, 44). Administration of activated protein C might be useful for maintaining an appropriate degree of immunothrombosis and preventing DIC. This is due to the fact that in sepsis, after thrombomodulin has been down-regulated by inflammatory cytokines, the conversion of protein C to activated protein C is suppressed (45).

In the clinical trial lead by the Recombinant Human Activated Protein C Woldwide Evaluation in Severe Sepsis (PROWESS) Study group, recombinant human activated protein C (rhAPC) was shown to reduce patient mortality from sepsis (46). However, a subgroup analysis also revealed that the administration of rhAPC was most effective in the severe group (those with an APACHE (acute physiology and chronic health evaluation) II score of 25 or higher). In fact, no reduction in mortality was observed in patients with an APACHE II score of less than 25, and the risk of serious bleeding increased in the patient group with APACHE II scores less than 20. In a metanalysis of the four RCTs that compared activated protein C versus placebo, the effectiveness of APC was not evident when a 28-day mortality was set as primary endpoint. Moreover, this metanalysis revealed the increased risk of bleeding associated with rhAPC (47). A ROWESS-SHOCK trial that focused on patient with septic shock found no significant beneficial effects on either mortality or bleeding (48). Ultimately, rhAPC was withdrawn from the market.

Thrombomodulin acts as dual regulator in coagulation and inflammation, making it a promising target for immunothrombosis in sepsis (49). In addition to generating an anticoagulant effect by converting protein C to activated protein C *via* the formation of a complex with thrombin, thrombomodulin exerts an anti-inflammatory effect by absorbing high-mobility group box 1 (HMGB-1) *via* the lectin-like region (D1) located at the N-terminus and by inhibiting leukocyte integrins *via* the serine/threonine domain (50, 51). Moreover, thrombomodulin binds to C3b and factor H, thereby negatively regulating the activation of C3b (52). Complement, which recruits and activates leukocytes, endothelial cells and platelets, is important to the innate immune system. However, its uncontrolled activation during sepsis can injure organs (53). Recombinant human soluble thrombomodulin (rhsTM) is an active extracellular domain comprised of thrombomodulin. To date, there have been many discussions regarding the clinical use of thrombomodulin for the treatment of septic patients. The SCARLET (Sepsis Coagulopathy Asahi Recombinant LE Thrombomodulin) trial that utilized rhsTM to treat sepsis failed to improve the 28-day all-cause mortality rate (54). At the moment, there is little clinical evidence to support the use of thrombomodulin to treat sepsis.

As discussed herein, many anti-inflammatory treatments targeting various pathways that have proven effective in animal studies have not yielded convincingly positive results in human sepsis clinical trials. Meanwhile, acknowledging that hyperinflammation is not the only key therapeutic target, the focus of sepsis research is shifting from only suppressing hyper-activated immune system to restoring paralyzed immune system.

### Paradoxical Immunoparalysis

Although sepsis has traditionally been characterized as constituting an aberrantly augmented immune response, the increasing prevalence of nosocomial infections in sepsis patients suggests the presence of sustained immunosuppression (55). The deregulations of the innate and adaptive immune systems are linked to the pathogenesis of the immunosuppression in sepsis (56).

Aberrantly induced cell death of immune cells represents a primary cause of immunosuppression in sepsis. A previous *in vivo* study utilizing Caspase-7 knockout mice have shown that lymphocyte apoptosis in sepsis was suppressed, which lead to the improved survival (57). Apoptosis is a highly regulated form of programmed cell death without eliciting an inflammatory response. Programed cell death and apoptosis are different in their details; for example, the caspase-dependent form constitutes apoptosis (58). There are classically at least two major signaling pathways that lead to apoptosis: the extrinsic pathway and the intrinsic pathway. The extrinsic pathway starts from binding ligands to those receptors anchored to cell membrane. Ligands are comprised of TNF, Fas ligand, and TRAIL (TNF-related apoptosis-inducing ligand). Receptors are comprised of TNFR-1, TNFR-2, Fas receptors (CD95), and TRAIL receptors. The triggering of these receptors by ligands results in the formation of a death-inducing signaling complex (DISC). Caspase 8 dissociates from the DISC to start the caspase activation cascade that lead to apoptosis (59). On the other hand, the intrinsic pathway is non-receptor-mediated; rather, it is driven by mitochondrias. Bax/Bak insertion into mitochondrial membrane causes cytochrome c release from the mitochondrial intermembrane space into the cytosol (60). Cytochrome c forms an apoptosome with Apaf–1 and procaspase-9. Apoptosome triggers caspase 9, which starts the caspase-3 signaling cascade toward apoptosis (61). Both the extrinsic and intrinsic pathways are activated and apoptosis-induced lymphopenia is observed in sepsis (62). Patients with sepsis exhibit apoptosis and/or suppressed functions of immune cells such as CD4+ and CD8+ T cells, B cells and dendritic cells (63) (64, 65). Among them, CD4+ helper T cells, which are crucial for directing appropriate immune responses, are divided into multiple subtypes: not only classically recognized Th1, Th2, and Th17 effector T cells, but also unique sub-populations such as regulatory T cells (Tregs) and Th9 (66). Th9 cells are an abundant source of IL-9, which is a pleiotropic cytokine that acts on many cell types. One immunological role played by IL-9 is the production of IL-4-mediated IgE and IgG from B cells (67). The Th1/Th2 paradigm cannot completely explain the role of current known helper T cells (68). Immune function is intricately suppressed by the apoptosis of various lymphocytes.

Unresponsive adaptive immunity caused by lymphocyte exhaustion is another mechanism of immuno-suppression in sepsis. In chronic viral infections and cancers, T cells are exposed to persistent antigens and inflammatory signals. Continued stimulation of T cells leads to gradual exhaustion. One of the mechanisms of exhaustion is the expression of PD-1 on the surface of T cells that function as immune checkpoints. Exhausted T cells lose their effector function. Specifically, they first lose IL-2 production and their high proliferative capacity, followed by a decrease in the production of IFNγ, TNF, and chemokines (69). The immune system is unable to mount an appropriate immune response against virus-infected cells or cancer cells. T-cell exhaustion is involved in the immune-paralysis in sepsis. T cells harvested from the spleen of patients who died of sepsis have only a low capacity to produce IFNγ and TNF, suggesting a state of T-cell exhaustion. This study also showed the increased expression of PD-1 on T cells and increased expression of PD-L1 on macrophages and endothelial cells (63). These results indicate the importance of the PD-1/PD-L1 pathway for lymphocyte exhaustion, eventually leading to poor outcomes in patients with sepsis. However, it is important to note that the activity of exhausted T cells can be restored by interfering with the PD-1/PD-L1 pathway, as described later.

Suppressed activities of antigen presentation is also responsible for immune suppression in sepsis. In sepsis, the expression of major histocompatibility complex (MHC) class II molecules and human leukocyte antigen DR isotype (HLA­DR) on antigen-presenting cells (APCs), including dendritic cells and macrophages, is reduced. In addition, the number of dendritic cells that undergo apoptosis increases in patients with sepsis (70). Decreased HLA-DR expression in monocytes is known to correlate with poor outcomes in sepsis (71). In animal studies, the impact of sepsis on tissue-resident dendritic cells differ in the systemic and mucosal organs (72) and the prevention of dendritic cell apoptosis improved the survival rate of sepsis (73). These facts support the contention that the reprogramming of antigen-presenting cells causes immunoparalysis in patients with sepsis.

The expansion of regulatory T cell and myeloid-derived suppressor cell (MDSC) populations constitutes an important mechanism to induce immunoparalysis in sepsis. Regulatory T cells usually maintain self-tolerance by suppressing the activation of auto-reactive effector T cells, thereby preventing auto-immune diseases under physiologic condition. Regulatory T cells increase during sepsis and generate pathologic immunosuppression by suppressing not only effector T cells, but also monocytes and neutrophil functionality (74). Inhibition of regulatory T cells has been reported to improve immunity and mortality rates in septic animal models (75). MDSCs are heterogeneous immature myeloid cells that are not normally detected, but which reach increased levels in cancers and sepsis (76). MDSCs suppress antigen-specific CD4+ and CD8+ T-cell activation (77). In addition, increased MDSCs are associated with greater numbers of regulatory T cells (78). Clinically, increased blood MDSCs have been linked to the increased prevalence of nosocomial infections in patients with sepsis (79).

### Immunomodulation; Past Failures and the Future Attempts

Recent progress in the sepsis research has revealed that immunosuppression occurs from the onset, but not in the late phase, of sepsis. The immune-suppression in sepsis results from the paralyzed immune system. The key to the treatment of sepsis is to restore the functions of the paralyzed immune system while avoiding the exacerbation of the immune system, which could induce unwanted hyperinflammation. In 1996, the restoration of the anti-tumor activity of T cells by CTLA-4 blockade was reported in a mouse model (80). A human lgG 1 kappa monoclonal antibody, ipilimumab, has been developed that binds to CTLA-4 (81). Therapeutic efficacy of Ipilimumab has been reported to correlate with the ability of receptors binding to Ipilimumab, indicating the importance of interaction between ligand and receptor of immune check point pathway (82). The clinical application of immunotherapy for cancer using checkpoint inhibitors targeting CTLA-4 and PD-1/PD-L1 pathway has been expanding. Many reports have been published on the safety of utilizing immunomodulation aiming to restore chronically suppressed immune functions in patients with malignant tumors (83). However, the immune suppression in patients with sepsis often occurs more acutely than in those with cancer. Moreover, patients with sepsis are severely ill requiring intensive care management compared with cancer patients. Thus, one should be cautious in extrapolating from the findings of the immune suppression in cancer patients to the application for sepsis patients.

A few growth factors have been studied to restore the impaired metabolism of the critical organ systems including the immune system in severely ill patients. Growth hormone administration has been reported to improve the balance of nitrogen in patients with sepsis (84). In surgical patients, the administration of growth hormones enhanced immunity by maintaining immunoglobulin levels, which led to the reduction of the postoperative surgical site infections (85). Growth hormone may restore the paralyzed immune system by maintaining protein synthesis in critically ill patients. However, in contrast to the expectation, two RCTs comparing high doses of growth hormone or placebo in critically ill patients showed increased rates of mortality. Moreover, growth hormone treatment was associated with prolonged mechanical ventilation, ICU stays and in-hospital admission days (86). These results fail to support the strategy of treating critically ill patients by improving protein synthesis with growth hormone. Similarly, Keratinocyte growth factor (KGF) aiming to restore the alveolar epithelial damages in the lung injury was studied in a Phase2 clinical trial for Acute Respiratory Distress Syndrome (ARDS) patients, thereby failing to improve their clinical outcomes. Moreover, KGF therapy was associated with the adverse events stemming from pyrexia (87).

Administrations of cytokines have been studied to restore the paralyzed immune system in sepsis. IFNγ has been shown to improve phagocytic capacity and HLA-DR expression on monocytes in human sepsis and related to earlier recovery from sepsis (88). Granulocyte-colony stimulating factor (G-CSF) and granulocyte-macrophage colony stimulating factor (GM-CSF) are also potential therapeutic agents for restoring the paralyzed innate immune system in sepsis. The meta-analysis performed in 2011 encompassing 12 RCTs revealed that G-CSF and GM-CSF failed to improve the mortality of sepsis patients (89). On the other hand, G-CSF and GM-CSF did not increase the frequency of complications related to hyperinflammation which was defined as life threatening organ dysfunctions like acute respiratory distress syndrome.

IL-7 plays a major role in the proliferation of CD4 + and CD8 + T cells, and also possesses antiapoptotic properties (90). Significant improvements in lymphocyte function by IL-7 have been observed in experiments involving an animal model of sepsis and using the blood of septic patients (91). The safety of IL-7 has been confirmed in clinical practice in patients with malignant tumors and HIV. In 2018, the phase 2b clinical trial studying the ability of recombinant human IL-7 to reverse the immunosuppression in sepsis demonstrated that the IL-7 treatment increased the number of lymphocytes without exacerbating inflammation (92). Future large-scale RCTs are expected to address clinical effectiveness of IL-7 for the treatment of sepsis.

## PD-1/PD-L as a Novel Therapeutic Target to Reverse Immune Paralysis

PD-1 represents a pivotal inhibitory checkpoint regulator that acts to dampen the activation signals elicited by T-cell receptor (93). PD-1 is a transmembrane protein widely expressed on immune cells including dendritic cells, NK cells and monocytes, which function as a costimulatory molecule (94). PD-L1 and PD-L2 are the ligands to which PD-1 binds and are expressed on the surface of various cells including tumor cells. PD-L2 binds to PD-1 with a higher affinity than does PD-L1 (95). The binding of PD-1 to PD-L suppresses T cell activation and cytokine production due to T-effector cell exhaustion and conversion of T-effector cells to regulatory T cells (96). By expressing the ligand of PD-1, tumor cells suppress the immune activity towards cancer cells, thereby escaping the elimination by immune cells. Several inhibitors to perturb the interactions between PD-1 and PD-L, thereby aiming to restore the paralyzed immunity against cancer, have proved clinically effective and been approved for the treatment of cancer (97). For example, Nivolumab, a human monoclonal antibody to PD-1, is used for the treatment of advanced melanoma (98). As a part of the mechanisms underlying immunosuppression in sepsis and malignancies are similar (99), the inhibition of the PD-1/PD-L1 interaction has been studied for theoretically restoring the immune suppressive states in sepsis.

### PD-1/PD-L in Sepsis

The enhanced expression of PD-L1 on various types of cells in sepsis has been documented. The expression of PD-L1 on both stromal cells and dendritic cells increased during sepsis. Splenic capillary endothelial cells from patients who died of sepsis expressed more PD-L1 than spleens from patients with brain death or trauma requiring emergent splenectomy (63). PD-1 is upregulated on T lymphocytes whereas PD-L1 is upregulated on monocytes in septic shock patients (100). The levels of PD-L1 expression on monocytes correlates with 28-day mortality rates in patients with sepsis (101). These findings support the idea that the aberrant activation of the PD-1/PD-L1 pathway constitutes a major cause of the immuneparalysis in septic patients.

### Clinical Trials Approaching for PD-1/PD-L

The significance of the PD-1/PD-L1 pathway in the pathogenesis of sepsis-induced immune paralysis has been substantiated in mouse sepsis models. Huang et al. have demonstrated that the survival rates of PD-1 knockout mice improved in a sepsis mouse model induced by the cecal ligation-and-puncture procedure, which caused bacterial pan-peritonitis leading to sepsis (102). Following up on this study, sepsis animal models induced by both bacteria and fungi have been treated with PD-1 or PD-L antibodies, improving overall survival rates (103–105), which support further evaluation in clinical trials. Nivolumab is the anti-PD-1 antibody approved for the treatment of cancer patients. The 2019 phase 1b clinical trial has studied the safety and tolerability of nivolumab given to septic patients, thereby showing that no adverse incidences including the aforementioned “cytokine storm”, were found (106). Another 2019 phase 1b clinical trial studying the safety of the anti-PD-L1 antibody BMS-936559 has also confirmed the safety in sepsis patients, thereby showing no adverse incidence of hypercytokinemia (107). Phase 2/3 clinical trials in the future are required to validate the PD-1/PD-L1 pathway as the therapeutic target for reversing the immune paralysis in sepsis.

### PD-1/PD-L and Innate Lymphoid Cells; Not Causing Immune-Paralysis But Rather Hyperinflammation

PD-1 on T cells has been well characterized and validated for the therapeutic target for cancer immunotherapy. A few recent investigations have studied the roles of PD-1 expressed on innate lymphoid cells (ILCs), emerging types of non-T, non-B lymphocytes lacking the expression of antigen receptors (108). ILCs play an important role both in homeostasis and in the inflammatory response in the immune system (109). ILCs are classified into at least three major subsets: type 1, type 2, and type 3 ILCs. ILC1, ILC2, and ILC3 execute the important immune regulatory roles to counterpart with Th (CD4+ T helper) 1, Th2, and Th17 T lymphocyte effector subsets, respectively. The roles of ILC2s in the lung inflammation induced by sepsis have been reported. ILC2, the most abundant ILC subset in the lungs, secretes type 2 cytokines such as IL-5 and IL-13, thereby playing the important roles in the regulation of type 2 immune responses, which is required for resolving inflammation and remodeling tissues (110). Insufficient resolution of inflammation and aberrant regulation of tissue repair and remodeling causes acute lung injury (ALI), and lung fibrosis, leading to the irreversible destruction of pulmonary functions. Thus, balanced regulation of ILC2 activation is critical for the treatment of sepsis, especially sepsis-induced pulmonary inflammation and ARDS.

Akama et al. investigated temporal changes in ILC2 functionality in the lungs of septic mice over time by examining the relationship of the ILC2 functions and the levels of lung injury in a cecum ligation and puncture (CLP) mouse sepsis model (111). The authors studied how the activities of ILC2s to produce the type 2 cytokine IL-13 correlated with the expressions of the stimulatory receptor ST2 and inhibitory receptor PD-1. IL-13 could exert a protective effect against sepsis by suppressing local inflammation, as shown by the previous study that antibody-mediated inhibition of IL-13 in the sepsis model increased macrophage inflammatory protein-2, macrophage inflammatory protein-1α and TNF-α levels (112). IL-33 is known to activate ILC-2 *via* ST2 receptor, thereby inducing the secretion of IL-13 that would promote the differentiation of macrophages to the anti-inflammatory M2 phenotype (113). The study has revealed that down-regulation of IL-13 in ILC2s correlated with the elevated expression of PD-1 on ILC2 in septic lungs, thereby suggesting that the PD-1/PD-L1 pathway in ILC2s functions as the inhibitory circuit to blunt type 2 immune response. IL-33 released from the injured pulmonary epithelial cells is necessary for the activation of ILC2 *via* the ST2 receptor. The induction of the type 2 immune response in a timely manner is critical for the resolution of inflammation, as shown by the experiment using IL-33 KO mice that lack the ILC2 activation (111). IL-33 KO mice showed delayed recovery from sepsis-induced systemic inflammation and wasting condition. Taken together, the perturbation of the PD-1/PD-L pathway in ILC2 could inhibit the induction of type 2 immune response in sepsis, thereby potentially compromising the resolution of inflammation.

### PD-1 and PD-L1 on Exosomes

The inhibitory signals through PD-1 on T cells are usually elicited by the binding to PD-L1 present on the opposing cells. Recent investigations have revealed that exosomes function not only as an alternative vehicle of PD-L1 to induce signals through PD-1, but also as a specialized platform to induce more robust inhibitory signals. EVs are lipid bilayered nanoparticles that contain RNA, DNA and proteins, thereby playing a role in intracellular communication. It has been reported that the depletion of exosomal PD-L1 by a genetic manipulation reversed cancer-associated immune suppression despite the intact presence of PD-L1 on the cell surface (114). The depletion of exosomal PD-L1 also inhibits tumor growth and achieve survival in a mouse model, supporting the idea that it is exosomal but not cell surface PD-L1 that is responsible for PD-1-mediated immune suppression. Of note, exosomal PD-L1 appears to be resistant to anti-PD-L1 antibody blockades aimed to interfere with the interaction of PD-1 on T-cells with exosomal PD-L1 (114). Although underlying mechanisms are unclear, exosomes present deep in the core of the tumor microenvironment might be sequestered and unreachable from systemically administered antibodies.

The roles of exosomes in the pathogenesis of sepsis have attracted much attention (115). PD-L1 and PD-L2 on exosomes circulating in the plasma of sepsis patients have been investigated by Kawamoto et al. who revealed that the beta2 integrin and PD-L2 on exosomes increased levels during sepsis compared to non-septic SIRS and health volunteers (116). Whereas the levels of PD-L1 on exosomes did not change in sepsis, the amount of circulating soluble PD-L1 including exosomal PD-L1 increased in sepsis. The levels of soluble PD-L1 and the leukocytic beta2 integrin showed significant correlation to clinically defined organ dysfunctions such as kidney injuries. The pathological roles of exosomal PD-L1 and PD-L2 in sepsis-induced immune suppression warrant further investigations in the future.

## Heterogeneity of Sepsis Syndrome: How the Main Obstacle for Clinical Trials Could Be Solved by AI

As a deeper understanding of the pathology underlying sepsis is being gained in the laboratories, many experimental therapeutic (but not prophylactic) treatments for inflammation suppression and immunomodulation have been successful in animal sepsis models (23–25). However, none of such treatments have proven effective in controlled clinical trials. The reason why they have not been successful in clinical trials could be animal models not recapitulating human sepsis pathophysiology as well as the diverse pathophysiology of human sepsis resulting in the heterogenous patient populations. Most animal sepsis models are developed in genetically homogenous laboratory animal strains using relatively unified “inducers”, such as administration of an endotoxin or bacterial peritonitis by CLP. In clinical practice, patients’ genetic and social backgrounds as well as types of sepsis inducers vary vastly; individual medical histories, the triggering infection, and the clinical course vary greatly among patients. Nonetheless, because sepsis is in fact a syndrome defined as a life-threatening organ dysfunction caused by a dysregulated host response to infection, it is diagnosed based on clinical symptoms and medical history, and not on the underlying molecular mechanisms, which can involve a wide variety of pathologies. At present, although sepsis has not been formally classified based on the underlying type of pathology, the identification of specific sepsis subgroups that could respond to a certain treatment is under intense investigations using a new enabling technology, AI. The drawback for classification is that sepsis has a clinically rapid time course compared to malignant tumors and chronic diseases. As the “Hour-1 Bundle” (117) proposal sets forth, medical professionals need to formulate a treatment policy as soon as a patient is diagnosed with sepsis. If lifesaving is to be successful, it is particularly important to detect sepsis using broad diagnostic criteria and not to miss those patients who need intensive care resources. It is extremely difficult for even a skilled clinician to classify sepsis based on the limited information typically available at the beginning of treatment. AI may make this possible.

### Artificial Intelligence and Sepsis

Machine learning in big data analysis is a fast-growing area in recent years (118). Unlike traditional statistical analysis, the process of the optimized classification derived by machine learning, in particular that by deep learning, is not transparent at first glance and is difficult for humans to understand and interpret intuitively (119). Furthermore, deep learning models, which form a part of machine learning, use a deep neural network to create an optimal model from raw data and require less human guidance. For example, both the hypothesis and verification that low oxygen saturation is related to the over-expression of PD-L1 on monocytes, resulting in an impaired immune response during sepsis, is easy to understand from our clinical standpoint (120). It is assumed by many of us that these kinds of reasonable hypothesis-based research paradigms are representing the mainstream science and will continue to play some roles in the future. However, AI has already begun to change the framework of research on sepsis in various ways such as the realization of very early diagnosis and disease subgroup classification.

A very early diagnosis based on the unbiased prediction is where AI can surpass human physicians, thereby potentially innovating the management of sepsis. As mentioned above, in clinical practice, the injection of antibiotics cannot be delayed in patients with suspected sepsis. Patients with suspected sepsis must be given antibiotics that can adequately combat the causative organism and be collected blood cultures within 1 h (117). On the other hand, excessive administration of broad-spectrum antimicrobials can generate a hotbed for drug-resistant bacteria, which should be avoided from a public health perspective. A rapid, sensitive and specific diagnosis is desirable. It has been shown that an algorithm constructed by machine learning can recognize sepsis hours earlier than can be done by humans in clinical practice (121). In fact, an algorithm created by AI that can predict sepsis up to 48 h in advance has been reported (122). Achieving early diagnoses using multiple parameters is a capability unique to the innumerable calculations made possible by machines.

Predicting the deterioration of specific organ functions at an early stage is similarly a specialty of AI. Although early detection of acute kidney injury is difficult to detect as a clinical symptom, early detection of it reportedly leads to improved prognosis (123). In 2019, with the growing demand for early diagnosis of acute kidney injury, an AI was developed based on the data from more than 700,000 cases that could predict the onset of acute kidney injury requiring hemodialysis. Its reliability measured 90% or more (124). The AI algorithm developed by DeepMind, a subsidiary of Google, known for its successful development of AI capabilities AlphaGo and AlphaStar that exceed the human world champion level of a strategy board game Go. With the advent of deep learning, not only medical professionals and medical researchers but also information technology specialists and engineers/programmers/data scientists are becoming more involved in the advancement of medicine.

As with kidney injuries, AI can also be used to predict the onset of ARDS, which is a serious condition of the lungs (125, 126). Ventilation management is essential in severe ARDS and requires significant medical resources. As this has emerged as a major social problem with the COVID-19 (coronavirus disease 2019) pandemic, predicting the onset of ARDS is crucial to determining policies aiming to properly allocate the limited medical resources such as ventilators and ICU beds. In addition, deep learning, which has led to treatment strategies that can have made direct contributions to clinical practice, continues to advance (127). This study showed that AI could offer more reliable treatment strategies than human doctors. In one validation cohort, the mortality rate was lowest when the treatment strategy of the human clinician matched that proposed by AI.

**Figure 2:**
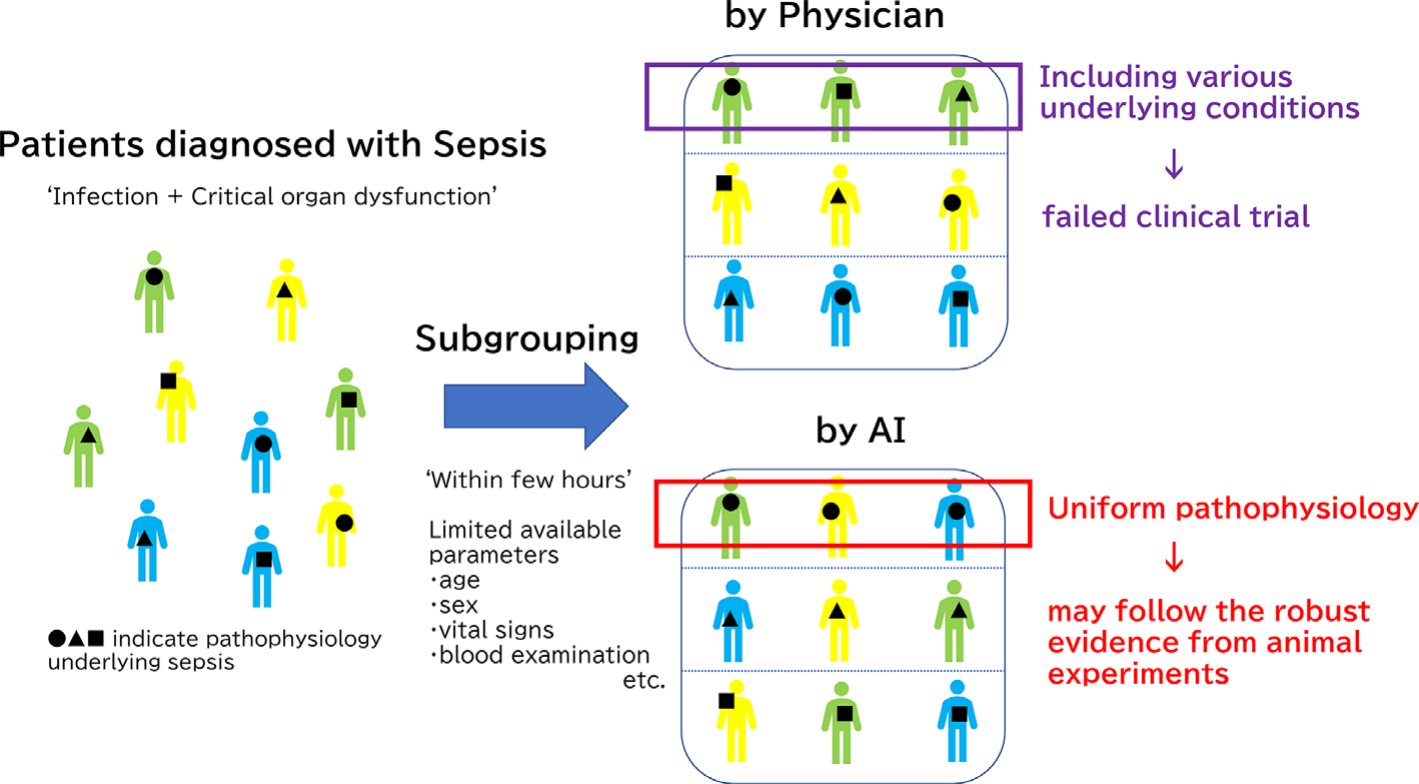
Subgrouping difference between human and Artificial Intelligence (AI). The reason why clinical trials have not been successful regardless of a deeper understanding of the pathology underlying sepsis is the diverse pathophysiology of sepsis in humans compared to animal sepsis models which are developed using relatively unified methods. Physicians usually classify septic patients by based on the types of primary disease, organ damage, and severity of systematic conditions that are consistent with clinical impressions. However, it is extremely difficult for even a skilled clinician to classify sepsis based on the limited information typically available at the beginning of treatment: medical history, vital signs, and few blood examinations. Thus, clinical trials could not have targeted specific patients. On the other hands, AI may achieve new classification, which is not transparent at first glance and is difficult for humans to understand, by machine learning. Only patients with immunoparalysis detected by AI should be treated with immune checkpoints inhibitors for successful clinical trials, for example.

In addition to the very early unbiased diagnosis of sepsis and organ failures, AI has made significant contributions to the clinically significant sub-grouping of sepsis. This could lead to the solution of the heterogeneity problem in sepsis, which underlies the difficulty of achieving translational success in the treatment of sepsis. In 2019, an innovative report was published in JAMA that sorted sepsis into four clinical phenotypes based on machine learning data analysis of 20,189 patients with sepsis (128). The raw data incorporated into this machine learning study were simply the parameters commonly collected through usual clinical practice: demographic information, laboratory abnormalities and organ dysfunctions. AI has achieved the unprecedented classification consisting of 4 sepsis subgroups: α, β, γ and δ that physicians have been unable to formulate on their own. The α phenotype shoewed neither abnormal blood results nor organ damage in α phenotype, thereby indicating the lowest in-hospital mortality rate. The β phenotype was older, had more chronic diseases, and suffered from renal dysfunction. The γ phenotype was marked by fever and elevated blood collection markers associated with inflammation and vascular endothelial damage. The δ phenotype showed the highest 28-day and 1-year mortality and hypotension and elevated serum lactate levels. Coagulation-related laboratory values were also notably elevated in this δ phenotype group. The four phenotypes of sepsis reported in the JAMA paper were different from the conventional classification system, which is based on the types of primary disease, organ damage, and severity of systematic conditions that are consistent with clinical impressions. (**Figure 2**)

As described earlier in this article, therapeutic agents used for immunothrombosis, such as activated protein C and thrombomodulin, failed to prove effective for sepsis in clinical trials (47, 48, 54). However, in the light of the AI-based new sub-group classification of sepsis, [e.g., α, β, γ, and δ phenotypes (128)], we may have to re-visit the experimental therapies. The formation of NETs is known to be involved in septic DIC, and the mechanism underlying neutrophil activation has been divided, in terms of molecular pathology, into two types; that by pathogen invasion and that by the indirect formation of suicidal NETs as a result of cytokine overproduction (129). The generation of NETs by the former mechanism can be suppressed by rhsTM (130). Even when the mechanism is examined at the molecular level, it remains impossible to recommend therapeutic agents based on the idea that sepsis is a singular condition. In other words, if the pathogenesis of NET can be inferred from clinical findings, clinical trials can be conducted only in those patients for whom rhsTM is effective (131). In this manner, results that might significantly improve patient prognosis could be obtained. It is reasonable to expect that AI will become capable of detecting cases of sepsis in which immunoparalysis is the main pathological condition, too. Targeting only the appropriate cases increases the likelihood that clinical trials will succeed.

AI has proven a powerful enabling technology in the management of sepsis, it is important to note that AI is not a panacea. The black-box problem and the frame problem are two major caveats that could potentially prevent the successful applications of AI in the field of medicine. The black box problem is that AI automatically makes an “optimized” decision for us without showing the process of optimization in such a way as human can understand intuitively. In the black-box model, we don’t usually have a clue as to whether the optimized decision made by AI is likely to be right or not. In an extreme scenario, we may not even know whether AI malfunctions as did HAL9000, a fictional AI in Arthur C Clarke’s Space Odyssey. In fact, malfunctioning of AI is not unreal in the face of the emergence of generative adversarial networks. While it is a merit of AI to be able to perform calculations that are not bound by human capacity and biases, applications of AI in medicine would demand a white-box aspect, in which the process of optimization is monitored and interpreted by human. Balancing interpretability and accuracy is an unresolved issue contemporarily. Further, the frame problem is an important one that has been pointed out for several decades (132). A myriad of events can happen in the real world, most of which have nothing to do with the immediate problem. A machine with only a finite amount of information processing power is not capable of dealing with all the possible real-world problems. AI related to sepsis has the limitation that a human must intervene, as it is necessary to specify which parameters should be used for learning. Fortunately, in the clinical setting of sepsis, the parameters available to the physician are limited to some demographic data, laboratory results, and vital signs due to time constraints. Therefore, realistically, almost all of these will be invested in the machine learning. However, creativity of adding more parameters, such as the addition of new blood tests, will still depend on humans.

## Discussion and Summary

Numerous clinical trials, including translational research based on animal experiments for sepsis, have been conducted. Nonetheless, there is still no clinically established treatment that targets the underlying essence of sepsis; i.e., the deregulated immune response leading to the simultaneous co-existence of hyperinflammation and immunosuppression. Reversing sepsis-induced immune suppression by inhibiting the PD-1/PD-L pathway could represent a promising therapeutic approach, awaiting a validation in clinical trials. A new consensus has also emerged that PD-1/PD-L is not merely the cause of T-cell exhaustion, but also affects ILCs. Moreover, rather than focusing on the expression of PD-L1 on immune cells, tackling exosomal forms of PD-L1 and soluble PD-L1 could lead to a breakthrough. The essential treatment for sepsis in the future will likely involve immunomodulation by inhibiting the PD-1/PD-L1 pathway in immunoparalyzed patients. Even though the immunoparalyzed subgroup of sepsis was not extracted in the previous machine learning study (128), it is important to identify such subgroups of immunoparalyzed. In the future, data-driven, AI-assisted personalized sepsis treatment might become a reality.

## Author Contributions

YN and MS contributed to the conceptualization, scope, and outline of this review. YN, EP, and MS analyzed the referenced manuscripts in this manuscript and participated in preparing the manuscript. All authors contributed to the article and approved the submitted version.

## Funding

This work was supported by the Japan Society for the Promotion of Science KAKENHI Grants (YN, 20K17835).

## Conflict of Interest

The authors declare that the research was conducted in the absence of any commercial or financial relationships that could be construed as a potential conflict of interest.
